# Robot Wars: US Empire and geopolitics in the robotic age

**DOI:** 10.1177/0967010617713157

**Published:** 2017-08-31

**Authors:** Ian GR Shaw

**Affiliations:** University of Glasgow, UK

**Keywords:** Drones, empire, geopolitics, robots, US military, warfare

## Abstract

How will the robot age transform warfare? What geopolitical futures are being imagined by the US military? This article constructs a robotic futurology to examine these crucial questions. Its central concern is how robots – driven by leaps in artificial intelligence and swarming – are rewiring the spaces and logics of US empire, warfare, and geopolitics. The article begins by building a more-than-human geopolitics to de-center the role of humans in conflict and foreground a *worldly* understanding of robots. The article then analyzes the idea of US empire, before speculating upon how and why robots are materializing new forms of proxy war. A three-part examination of the shifting spaces of US empire then follows: (1) *Swarm Wars* explores the implications of miniaturized drone swarming; (2) *Roboworld* investigates how robots are changing US military basing strategy and producing new topological spaces of violence; and (3) *The Autogenic Battle-Site* reveals how autonomous robots will produce emergent, technologically event-ful sites of security and violence – revolutionizing the battlespace. The conclusion reflects on the rise of a robotic US empire and its consequences for democracy.

## Introduction

Robots fascinate humans. Whether in dystopian tales of cyborg warriors or the growing anxieties about mass unemployment, the robot is a science-fiction archetype that haunts our cultural unconscious. But it is the military applications of robots that hold the potential for rewiring the old rules of geopolitics. This article examines the *robotic futures* that are being imagined and projected by US military officials, think-tanks, and defense documents (e.g. Defense Science Board, 2016; Scharre, 2014; US Air Force, 2016; US Department of Defense, 2013; Work and Brimley, 2014). These futures reveal profound developments in robotic warfare, artificial intelligence (AI), and drone swarming. Uniting these trajectories, this article analyzes how robots are transforming the spaces, logics, and geopolitics of US *empire.* While robot futurologies are now routinely constructed by defense officials and academics, ‘empire’ is rarely used as a heuristic. And yet, given ‘empire in some form has persisted over the millennia, and will likely continue into the foreseeable future’ (McCoy, 2012: 6), the term significantly enriches our geopolitical and conceptual understandings of the looming robot wars.

**Figure 1.**
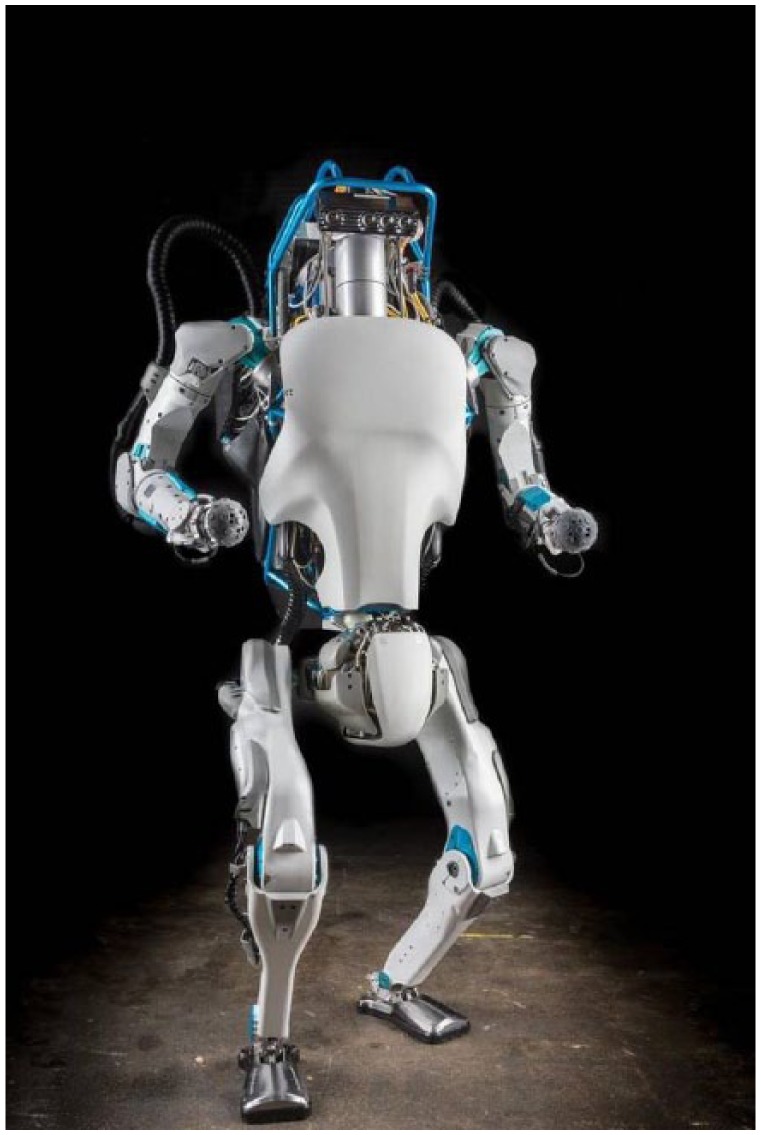
Boston Dynamics’ humanoid robot, Atlas (2016). *Source*: Wikimedia Commons: *https://commons.wikimedia.org/wiki/File:Atlas_from_boston_dynamics.jpg?uselang=en-gb.*

There is no single definition of what constitutes a robot. In fact, the very naming of a robot is a categorical act that bulldozes important differences. Nonetheless, a robot is typically defined as a computer-programmable machine capable of automatic actions. This encompasses a range of artificial devices: flying robots, such as the iconic Predator drone; humanoid robots, like Honda’s ASIMO or Boston Dynamics’ Atlas; the smart cars driving in San Francisco; or the industrial robots that work in factory floors across the planet. *I, Robot*, Isaac Asimov’s 1950 science-fiction classic, outlined the Three Laws of Robotics. These were designed to protect humanity against their robotic creations. The most important was the first law: A robot may not injure a human being or, through inaction, allow a human being to come to harm. Over 60 years later, a variety of US military robots of various shapes and sizes – such as the Reaper drone – are now engineered to directly violate Asimov’s law. For Peter Singer (2009: 194), this means ‘humans’ 5000-year-old monopoly over the fighting of war is over’.

In response to this growing robotic revolution, which takes place in and beyond military industrial complexes, US Deputy Secretary of Defense Robert O. Work warned that the US military ‘cannot afford to defer the time, thinking and investments needed to prepare for warfare in the Age of Robotics’ (Work and Brimley, 2014: 36). The robot age thus confronts militaries, politicians, scholars, scientists, and activists with deep questions about the future of warfare, and even our humanity. Across the suburbs, favelas, and cities of the globe, are we witnessing the rise of an expansive robotic battlespace, ‘a grim and grimy Terminator Planet’ (Turse, 2012a)?

At the heart of these futurologies lies a big uncertainty, one only glimpsed at with drone warfare today: How will the US military project its power in a multidimensional, multinational robotic age? To answer this question, the article turns to ‘empire’ – a concept that ‘has played little role in international relations theorising’ (Mabee, 2004: 1368; see also Stokes, 2005). ‘Empire’ is an important heuristic for situating this futurology: it embeds the robotic warscapes, infrastructures, and weapons of the future within a much longer geo-history. Indeed, for Noel Parker (2010: 114), empire ‘might still be the “Great Game” in global geopolitics’.

Empire continues to attract debate in international relations (Mabee, 2004; Munro, 2014). Academics on both the left and the right of the political spectrum have argued that the USA has been an empire since at least the Cold War (Johnson, 2004a). Others trace a post-9/11 history to describe the neoconservative Bush administration. As Michael Ignatieff (2003) wrote in 2003, ‘what word but “empire” describes the awesome thing that America is becoming?’ Yet the Bush-era counterinsurgencies in Iraq and Afghanistan have long ended. This drawdown was driven, in part, by the ascent of drone warfare under the Obama administration’s small-footprint approach to counter-terrorism. Nonhumans – from aerial robots to computer algorithms – have become increasingly responsible for managing the spaces of violence of what Derek Gregory (2011) terms an ‘everywhere war’.

Since the US Central Intelligence Agency (CIA) increased the number of Predator drone strikes under the Obama administration, there has been a surge of interest in remote warfare. Scholars have researched its historical (Kindervater, 2016; Satia, 2014), philosophical (Chamayou, 2015), legal (Shaw and Akhter, 2012), spatial (Gregory, 2014), ethical (Schwarz, 2016), and material dimensions (Holmqvist, 2013; Walters, 2014). Drone warfare continues to complicate the relationship between humans and nonhumans (Wilcox, 2017), as well as the status of sovereignty and territory (Shaw, 2016). Yet current drone warfare is just the beginning of a robotic revolution sweeping across the military: future drones will be smaller, autonomous, and capable of interacting in swarms. And these autonomous leaps ‘have the potential to fundamentally change the American way of war’ (Sukman, 2015: 44). Robots, whether in the air, on the ground, or underwater, are not epiphenomenal to the future of US empire, but enmeshed in its composition – and the warscapes it will secure.

Accordingly, this article advances a futurology for understanding how robots will materialize a robotic US imperium. ‘With an agile force directed via a robotic information infrastructure’, argues McCoy (2012: 384–385), ‘the United States could, in principle, parlay its military power into a second American century … creating something akin to an endless American empire.’ This endless empire depends on how the US military pivots towards a robot-intensive form of counter-terrorism. To understand this pivot, we require an understanding of robots as actors that transform the materiality of our more-than-human (security) worlds. In making this argument, the article contributes to research in international relations, geopolitics, and political geography on the materiality of world politics and state power (e.g. Aradau, 2010; Bourne, 2012; Cudworth and Hobden, 2013; Meehan et al., 2013; Schouten, 2013; Walters, 2014; Wilcox, 2017). Of course, the futurology constructed in this article, like all futurologies, is necessarily contingent, fragile, and non-linear.

The rest of the article is structured as follows. First, the article outlines a more-than-human geopolitics to de-center the role of humans in conflict and argue for the importance of a worldly understanding of robots. It then examines the robotic revolution sweeping across the US military. After that, the article analyzes the history of US empire, before speculating upon how and why robots are materializing new forms of proxy warfare. A three-part examination of the changing spatialities of US empire then follows: (1) *Swarm Wars* explores the implications of miniaturized drone swarming; (2) *Roboworld* investigates how robots are changing US military basing strategy and producing new topological spaces of violence; and (3) *The Autogenic Battle-Site* reveals how autonomous robots will produce emergent, technologically event-ful sites of security and violence – thereby revolutionizing the battlespace. The conclusion reflects on the rise of a robotic US empire and its consequences for democracy.

## Robot worlds: A more-than-human geopolitics

In this section, I make the case for why geopolitics is important for understanding the *worldly* dimensions of the robotic revolution. After making this general point, I outline the more specific concept of a more-than-human geopolitics. Critical security studies, political geography, and international relations scholars have made important interventions in examining materiality and state power (e.g. Bourne, 2012; Cudworth and Holden, 2013; Dittmer, 2013; Schouten, 2013; Shaw and Akhter, 2012, 2014; Walters, 2014; Wilcox, 2017). This attention to materiality is intersected by Deleuzian-inspired accounts of race, religion, gender, nationality, and political affect (e.g. Protevi, 2009). Notwithstanding these contributions, this section discusses geopolitics – a concept that helps foreground the spatiality of the robotic revolution. While the relationship between matter and society has long been studied – particularly in the Marxist tradition – geopolitics foregrounds the *worldliness* of power, politics, and matter. Human life, writes Hannah Arendt (2013: 95–96), is ‘world-building’. So, whether tank, radio, nuclear weapon, or drone, each has changed the composition of our geopolitical worlds and the spatiality of human coexistence.

Indeed, ever since humans first crafted stone instruments, we have existed as *tool-beings*, building worlds big and small, extending our biological lives, our ability to communicate, and our ability to harm, outwards in time and space. Technology is not merely a passive vessel, then, but enables, limits, and redefines the ontological conditions for worlds. Yet, when it comes to discussions of robots, an underlying realism often dominates mainstream and academic discussions. The robot is viewed as a machine that is manipulated *by* humans *for* humans. Accordingly, we need to challenge the *realist*, *aspatial*, and *apolitical* assumptions of our robotic condition. Robots are existential actors precisely because they secure, reconstruct, and at times destroy the worldliness of human coexistence. The concept of geopolitics equips us with the conceptual tools to explore these worldly futures.

Classic geopolitics, born in the midst of European imperialism, studied how the physical topography of the planet conditioned foreign policy (see Dittmer and Sharp, 2014). Yet the nonhuman landscape was rendered neutral, objective, and, most importantly, separate from human agency. This dichotomy remains active in realist branches of international relations, with the material world viewed as *exogenous* to society and *instrumental* to human intentionality (Bourne, 2012). Claudia Aradau (2010: 493), for example, argues that ‘non-human objects have been relegated outside the realm of securitization’. Or, as Peer Schouten (2013: 563) argues, international relations ‘has been oblivious to the significance of material civilisation for the constitution and transformation of political order’. The consequence of treating the nonhuman as a mere vessel of power is that empire, warfare, and geopolitics are restricted to an overriding humanism, where ‘the only ordering agent on the scene is *people*, in the form of the sovereign human’ (Harman, 2014: 18, emphasis in original).

A more-than-human geopolitics, conversely, incorporates the dynamic agency of humans *and* nonhumans, as well as the assemblages by which state power is transformed (Dittmer, 2013). Accordingly, this framework foregrounds the artificial, cyborgian, and algorithmic materializations of state power in the world system. More specifically, exponential leaps made in artificial intelligence, machine learning, and the sophisticated robots that embody these algorithmic instructions all demand a reflection into – even revaluation of – the ‘who’ and the ‘what’ of warfare. Robotic intelligence is likely to figure centrally in the crises, discontents, and conflicts of the future world system.

A more-than-human geopolitics thus moves from a subjective model of power to incorporate insights from *techno-politics*, ‘a concept that captures the hybrid forms of power embedded in technological artifacts, systems, and practices’ (Hecht, 2011: 3). Through this challenge to human-centered accounts, ‘objects are suddenly highlighted not only as being full-blown actors, but also as what explains the contrasted landscape we started with, the over-arching powers of society, the huge asymmetries, the crushing exercise of power’ (Latour, 2005: 72). Political geographers, for example, have defined the state as an assemblage of objects, ‘considering “force relations” as the primary field through which a whole manner of different objects, bodies, and doings are policed’ (Meehan et al., 2013: 8; see also Shaw and Akhter, 2012, 2014). Nonhumans – whether AK-47s, computer viruses, drones, or barbed wire – are motor engines of force that police, patrol, and secure the world system. As Schouten (2013: 559) writes, politics ‘is in the first place the result of introducing non-human entities that give durability and “body” to social arrangements’.

This act of technical translation is inseparable from human subjectivity (see Wilcox, 2017). For Hannah Arendt (2013: 9), ‘the objectivity of the world – its object- or thing-character – and the human condition supplement each other’. Accordingly, it is vital to see objects and subjects as intertwined forces that crystalize in the *materialization* and *securitization* of worlds (Aradau, 2010: 494). What we consider as ‘reality’ is not a neutral plane of existence but a contorted landscape of artificial and hyper-secured worlds. But these worlds are never fully under our control: nonhumans continually materialize unexpected geographies. Understood in this way, robots are not passive instruments but existential actors capable of reprogramming the worlds of human coexistence. Accordingly, the shift from a human to a more-than-human geopolitics does not signal the end of the political: it sharpens our tools for understanding the ontological asymmetries and injustices of worlds.

## The robotic revolution

Robots have disrupted the social and economic spaces of human coexistence for decades. In 1961, General Motors installed the first industrial robotic arm – *Unimate* – on its assembly lines in New Jersey. Since then, robots have migrated from factories to the social spaces of everyday life. According to one report, there are over 8.4 million robots globally, with a market value in excess of $15 billion (Kadtke and Wells, 2014: 43). And with the exponential leaps in microprocessing power predicted by Moore’s Law, we now stand on the verge of a ‘Cambrian’ explosion of robotic life on planet earth. This has big consequences for the spaces of security and violence. On the ground, in the air, and underwater, military robots are eroding the human monopoly on violence. ‘Today, the world is approaching a robotics revolution in military affairs that may be on par with the introduction of gun-powder, *levée en masse*, and the advent of nuclear weapons’ (Sukman, 2015: 44).

The coming decades will see US soldiers augmented, replaced, and wounded by artificial warriors, which ‘has the potential to change our basic core concepts of defense strategy’ (Work and Brimley, 2014: 6). Yet the robotic revolution must be placed within a longer trajectory of capital-intensive warfare (see Edgerton, 1991). This liberal form of violence aims to use technological superiority to ‘solve’ the problem of ‘uncivilized’ states and actors (Mabee, 2016). Accordingly, the US military continues to project *peace through capital, security through technics.* In 2014, the USA accounted for just over half of global arms sales in a $70 billion annual industry (Theohary, 2015). Still, robots are disrupting the architectures of capital-intensive warfare. The future-oriented *20YY: Preparing for War in the Robotic Age* sheds light on the US military’s production of robotic geopolitical futures. Co-authored by Deputy Secretary of Defense Robert O. Work, it insists that ‘U.S. defense leaders should begin to prepare … for war in the Robotic Age’ (Work and Brimley, 2014: 5). A similar report by the Pentagon-funded National Defense University explores how evolutions in synthetic life, robotics, and artificial intelligence will transform US dominance (Kadtke and Wells, 2014).

**Figure 2.**
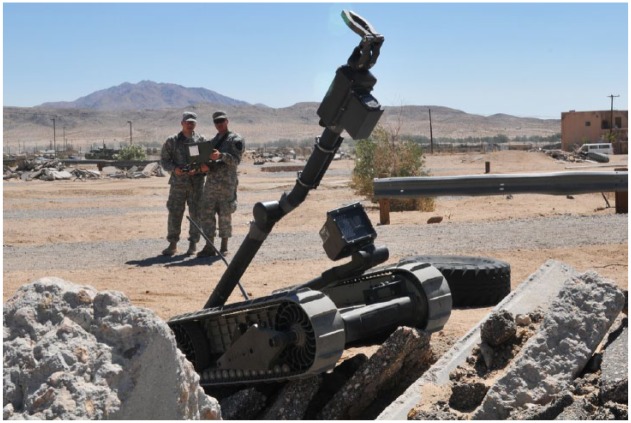
iRobot’s Packbot at the US National Training Center. *Source*: US Army photo by Sgt Kimberly Hackbarth. Image from Wikimedia Commons: *https://commons.wikimedia.org/wiki/PackBot#/media/File:Flickr_-_The_U.S._Army_-_Robotics_class.jpg.*

At the outset of the invasion of Iraq, the US military had zero ground robots. By the end of 2008, there were 12,000, including iRobot’s PackBot, which was integral to counter–improvised explosive device (IED) missions. Aerial robots continue to disrupt the spaces, subjects, and geopolitics of US conflict (Shaw, 2016). Indeed, what Kate Kindervater (2016) calls ‘lethal surveillance’ has transformed US geopolitics. The Pentagon now stocks a fleet of well over 12,000 aerial drones (US Department of Defense, 2013). Controversially, medium-altitude Predator and Reaper drones have been deployed outside of ‘hot’ battlefields in Pakistan, Yemen, and Somalia (Shaw and Akhter, 2012). And on a smaller scale, the US Army is researching how to equip its soldiers with tiny pocket-sized drones for ‘over the hill’ surveillance, which could revolutionize infantry tactics. Within the USA, Predators and Reapers are routinely flown by US Customs and Border Patrol along the Mexico and Canadian borderlands. And back on the ground, $55 million has been spent on transferring 1000 military ground robots to police forces across 43 US states (Center for the Study of the Drone, 2016). On 7 July 2016, Dallas Police Department became the first to use an exploding robot to kill an armed suspect. A robot red-letter day.

How, then, can we piece together these developments? Former secretary of defense Chuck Hagel unveiled the Defense Innovation Initiative, also known as the ‘third offset strategy’, on 15 November 2014. Underpinned by robotics, cyber warfare, autonomy, 3D printing, electric weapons, miniaturization, and the internet of things, this strategy is designed to sustain US technological dominance in the 21st century. The ‘third offset’ follows precedents in the Cold War. To offset Soviet numerical advantages in the 1950s, the US military invested heavily in nuclear weapons. The second offset strategy of the 1970s was driven by stealth, the Global Positioning System (GPS), and network-centric systems. In both cases, technology was translated into US military dominance. Yet, unlike in the Cold War, the robotic revolution today is sweeping across multiple nations (and non-state actors), not just the US and Soviet military-industrial complexes. Thus, US empire confronts a robotizing warscape that is both global and unpredictable.

## US empire: Towards a proxy army of robots

In the preface to the 2015 *National Security Strategy*, US President Barack Obama declared, ‘We possess a military whose might, technology, and geostrategic reach is unrivalled in human history’ (White House, 2015). Rarely, however, is ‘empire’ used in official parlance. Yet, as Mabee (2004: 1360) suggests, ‘while “lonely superpower”, “unipolar”, and other monikers have been utilized, the US as empire seems to convey a richer understanding and a deeper resonance of America’s contemporary role’. But what exactly *is* empire? ‘Empires are usually states’, replies Zielonka (2012: 519), ‘but they are states of a peculiar nature.’ Can a state-centric understanding capture the global infrastructures of US military power? For Dalby (2005: 435), ‘If empire is to be of analytical use, it seems that none of the versions of it in circulation are quite adequate to grapple with nuances of the present.’ Accordingly, the concept of empire must connect the geographies of contemporary technics with past materializations. As Parker (2010: 128) concludes, ‘for all its differences from nineteenth- and twentieth-century examples, the geopolitics of empires is likely to be found in the present and in the future’.

US history is inscribed by imperialism. For Munro (2014: 1567), ‘continuing to think of US history in imperial terms enables us to see how the present arrives already fundamentally shaped by past economic disparity, racial inequality, hetero-patriarchal oppression and a deep interrelation between the realms of foreign and domestic’. Accordingly, Heumann (2011) argues that the USA has always been an *empire-state* rather than a *nation-state*, inheriting the precedents of European empires. Practices of racial domination and expansionism underwrote US state formation and early capitalism, leaving a legacy of segregation. Fast forward to the 19th century, and the so-called Monroe Doctrine of 1823 articulated the belief that the USA was a guardian against European intervention in the ‘New World’. Under President Woodrow Wilson, this moral imperative was reworked into an idealistic vision for spreading democracy across the planet. The grim parade of US proxy wars and black ops during the Cold War – particularly in Latin America – entrenched the idea that the USA was the guardian of western civilization.

Despite this history of foreign intervention, the USA has consistently escaped the label of empire (Johnson, 2004a). This did change during the ‘war on terror’, as the Bush administration adopted ‘a new ideological commitment to empire’ (Agnew, 2003: 883). Consider the now infamous boast of White House adviser Karl Rove: ‘We’re an empire now, and when we act, we create our own reality’ (originally quoted in Suskind, 2004). Supporters of US empire ranged from liberal interventionists to neoconservative hawks. Under both visions, ‘the US state is thus seen as an imperial state overseeing a global empire which brings benefits to both other Western states and also the inhabitants of war-torn states’ (Stokes, 2005: 219). Or, as Michael Ignatieff (2003) declared, ‘The 21st century imperium is a new invention in the annals of political science, an empire lite, a global hegemony whose grace notes are free markets, human rights and democracy, enforced by the most awesome military power the world has ever known.’

Others find the idea of US empire implausible in an era of global capital (Hardt and Negri, 2000: 384). Empire is viewed instead as a deterritorialized form of capitalist world sovereignty. Yet globalization should not imply a flat and frictionless world of capital. The USA has long been the leader of an *uneven* world capitalism since the Bretton Woods agreement of 1944. Accordingly, ‘there is a geography of globalisation: one that rests on an imperial centre’ (Mabee, 2004: 1360). Thus, while capitalist logics are vital in explaining geopolitics, so too are the planetary materialities of US empire. Empires entrench their power through technics. No human sovereign is as awe-inspiring as the technical background that secures humanity across the planet. The question then becomes, how will US empire materialize – and maintain – its dominance in an age of global robotics, technological diffusion, and powerful non-state actors? If the third offset strategy is an indication, US empire will reconstitute by shifting to a permanent, robotic *proxy war.*

Since World War II, the practice of total warfare between states has diminished and proxy wars have increased (Kaldor, 2012). One of the most infamous examples in US history was the CIA-funded Afghan mujahedeen in the 1980s. More recently, the Pentagon spent billions on private military contractors in Iraq. But rather than outsource military power to human contractors, US military surrogates of the future could be replaced by robots: private military contractors without flesh, risk, or vulnerability (or healthcare costs). As Mumford (2013: 43) suggests, ‘developments in communications and information technology have the potential to nullify the twentieth-century belief in “boots on the ground” as a proxy-war necessity’. An army of robots can entrench US power without humans on the ground. And, just as importantly, these artificial warriors are *profitable* commodities (Shaw, 2017). A robotic US empire thus advances proxy warfare – and capitalism – to its most logical conclusion.

## Autonomous futures

Autonomy – and AI more generally – is central to the future of US geopolitics. The Defense Science Board (2016: 3) writes that the ‘ongoing rapid transition of autonomy into warfighting capabilities is vital if the U.S. is to sustain military advantage’. Unlike automation, an autonomous system possesses adaptable intelligence. This enables it to be ‘goal-directed in unpredictable environments and situations’ (US Air Force, 2016: 30). Autonomous weapons are not entirely new: since the 1980s the US Navy has installed autonomous Phalanx CIWS Gatling Guns on many of its battleships. Nonetheless, the contemporary warscape is being transformed by ‘an emerging class of robotic weapons, including drones, mobile sea mines, automated turrets, remote-controlled machine guns, as well as weaponized computer programs’ (Bolton, 2015: 41). Several autonomous drones – the US Navy’s MQ-8 Fire Scout, the UK’s Taranis drones, and the Anglo-French Unmanned Combat Air System – promise to revolutionize air combat. Fearing this geopolitical pathway, 1000 leading scientists called for a ban on autonomous robotics in 2015, citing the existential risks to humanity. Similarly, Human Rights Watch and the International Human Rights Clinic (2016) continue to demand the prohibition of what they term ‘killer robots’.

The US military regularly produces roadmaps that imagine autonomous futures: from robots extracting causalities to swarms of drones overwhelming enemies. ‘Autonomy in unmanned systems will be critical to future conflicts that will be fought and won with technology’ (US Department of Defense, 2013: 67). Multiple reasons are given to justify the Pentagon’s quest for autonomy: future threats will be too complex for human reactions, autonomous robots can function if they lose radio contact, and using pilots to oversee multiple robots is more cost-effective. Finally, autonomous systems can analyze huge streams of real-time data ‘in ways that humans cannot by addressing volume, complexity, speed, and continuity’ (Defense Science Board, 2016: 47). Autonomy thus enables robots ‘to make and execute complex decisions’ (US Army, 2010: 65). Future pilots would direct swarms of intelligent drones with a geographic information system (GIS) program: *on the loop* but not *in the loop.* ‘The end result would be a revolution in the roles of humans in air warfare’ (US Air Force, 2009: 50).

This standard of AI enables future drones to break with the restrictions of existing unmanned systems. ‘Future [unmanned aircraft] will evolve from being robots operated at a distance to independent robots, able to self-actualize to perform a given task’ (US Department of Defense, 2005: 52). This self-actualization enables the evolution from autonomous targeting to the more controversial step of autonomous attacking (US Department of Defense, 2007: 54). Yet, while the US military is a world leader in robotics today, it will face future ‘unknown unknowns’ from other militaries and non-state groups. For example, there is growing concern within the Pentagon that US soldiers are now vulnerable to (swarms of) terrorist drones on the battlefield. Accordingly, the drone-on-drone warfare of the future will produce highly contested airspaces, fueling a robot arms race of offensive and defensive systems.

Autonomy, however, is more than just artificial intelligence: it is an ontological condition. Robots, like all technologies, materialize unique techno-geographies and techno-politics (Hecht, 2011). Rather than being slavish instruments of human minds, robots are geopolitical actors that disrupt and reinvent the worldly conditions of state power, installing ontological coordinates – predictable and unpredictable – for future military violence. The epistemological problem of *if*, or *how*, we go to war is inseparable from background material conditions. Drones, for example, continue to transform the ethics of targeted killing (see Walters, 2014). ‘Drones enable the (de)politicization of targets by abstracting human life into a techno-political entity that can be captured in clinical terms as data’ (Schwarz, 2016: 61). So, while robots do not straightforwardly *determine* military operations, they nonetheless *recondition* their field of possibilities. ‘Robots, then, may not only make it easier to start a war … they may actually change our military and political doctrines and activities’ (Coeckelbergh, 2011: 271). In short, robots always already possess a degree of ontological autonomy because they reconfigure the conditions of a more-than-human warscape.

## New spaces of empire I: Swarm wars

Autonomy enables the next change in US warfare: *swarming*, which holds the potential for ‘rendering previous methods of warfare obsolete’ (Work and Brimley, 2014: 29). The US military is at the beginning of an ontology of war that shifts power projection from discrete platforms (such as expensive fighter jets and aircraft carriers) to amorphous and autonomous swarms. The military defines swarming as ‘a group of autonomous networked [small unmanned aircraft systems] operating collaboratively to achieve common objectives’ (US Air Force, 2016: 43). In this sense, the Predator family of drones is the harbinger of a miniaturized warfighting regime. US empire will go further by going smaller. The hugely popular (and hand-launched) Raven drone embodies the revolutionary aspect of future robotic violence: smaller units and payloads. Indeed, researchers at Harvard have developed ultra-cheap 3D-printed drones that ‘could allow the United States to field billions – yes, *billions* – of tiny, insect-like drones’ (Scharre, 2014: 20). Consequently, military power and vulnerability are dispersed, overturning individual survivability with swarm resiliency. Such a quantitative change in the number and size of military robots will install a qualitative shift in the warscape.

Swarming advances the network-centric warfare popularized in the 1990s. In their RAND report, John Arquilla and David Ronfeldt (2000) place swarming at the center of future US military strategy. Swarming embodies a movement from the *network-space* of the second offset strategy to the *swarm-space* of the third offset, which ‘will reshape the future of conflict as surely as the rise of blitzkrieg altered the face of modern war’ (Arquilla and Ronfeldt, 2000: 3). Just as swarming insects are not particularly intelligent, cheap, swarming robots can collectively perform complex tasks with simple algorithms. Swarms of amorphous drones are thus ideal for overwhelming a nonlinear battlespace, ‘creating a focused, relentless, and scaled attack’ (US Air Force, 2009: 16), using ‘a deliberately structured, coordinated, strategic way to strike from all directions’ (Arquilla and Ronfeldt, 2000: vii). And perhaps the only way to defend against such a swarm would be with a more sophisticated swarm. ‘The result will be a paradigm shift in warfare where mass once again becomes a decisive factor on the battlefield’ (Scharre, 2014: 10). Previous offset strategies substituted mass with precision weapons. The return to mass as a medium of military power is, however, different from the past. Mass in the 21st century requires a *molecular* and *plastic* robotic mass: one that mirrors the swarms of bees, fish, ants, and birds in the natural world.

**Figure 3.**
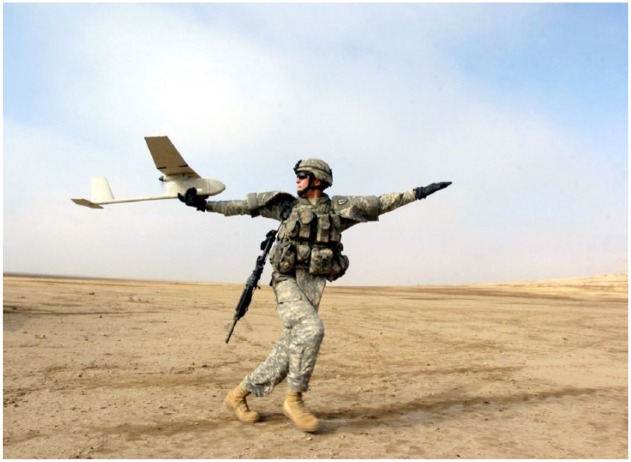
RQ-11 Raven. Photo by Sgt 1st Class Michael Guillory (2006). *Source*: Wikimedia Commons: *https://commons.wikimedia.org/wiki/Unmanned_aerial_vehicle?uselang=en-gb#/media/File:RQ-11_Raven_1.jpg.*

Swarming thus materializes a nonlinear swarm-space: a massed atmospheric attack. Targets are secured and overwhelmed by intelligent drones acting and moving faster than humans. This shifts the battle-regime from the surfaces of land power and the skies of air power, to the swarm-spaces of robot power, crystallizing a volumetric and multidimensional geometry of violence. This upturns the spatial pointillism and logic of human control in current drone warfare. First, swarming drones will move in emergent and self-cooperating groups. Here, distinct targets and static geographies collapse: the weapon is swarm-space itself. Second, the causal link between pilot and drone is transformed into an emergent rule-set, in which swarms are directed by onboard artificial intelligence. Third, swarms will interact across the spectrum of military domains (terrestrial, maritime, cyber, and outer space), part of what the military calls full spectrum dominance.

Swarm-space could infest small corridors, passageways, and urban volumes that were previously inaccessible to medium-altitude drones. As the US Army (2010: 65) foresees, ‘swarms will have a level of autonomy and self-awareness that will allow them to … fly, crawl, adjust their positions, and navigate increasingly confined spaces’. DARPA’s Fast Lightweight Autonomy program is an example of an emerging class of algorithms being developed to enable robot swarms to operate in cluttered urban environments. Thus, robotic swarms – both military and non-military – could inflict massive damage across cities in the global north and south, and install new regimes of intimate and diffuse surveillance (Shaw, 2017). Indeed, in so many ways, swarming already mirrors the post-Fordist disaggregation of society that no longer functions in bounded and linear aggregates (such as classes, families, and communities), but in ephemeral, dislocated groups. The modernist barriers between people, things, and places are dissolving into swarms, waves, and foams (Sloterdijk, 2013).

## New spaces of empire II: Roboworld

US empire in the robotic age will continue to project technical power in lieu of corporeal vulnerability. Already, the reliance on drone warfare has transformed the geography of overseas military basing. Chalmers Johnson (2004b) wrote that the 20th century saw the US military entrench a ‘globe-girdling Baseworld’. In the Cold War, around 1700 US bases were constructed across Europe and the Pacific to ‘contain’ communism. As a result, argues Johnson (2004a: 188), US empire is ‘solely an empire of bases, not of territories, and these bases now encircle the earth in a way that, despite centuries-old dreams of global domination, would previously have been inconceivable’. The Pentagon lists 523 military bases, part of a property portfolio of 562,000 facilities across 4800 worldwide sites, covering 24.7 million acres (US Department of Defense, 2014). In 2015, David Vine estimated there were closer to 800 US bases operating in 80 different countries, costing $165 billion a year. As Vine (2015) writes, ‘the United States likely has more bases in foreign lands than any other people, nation, or empire in history’.

The drone has disrupted the military necessity to house soldiers abroad. By 2016, the Predator family of drones – Predator, Reaper, and Gray Eagle – had flown over 4 million flight hours over 291,331 missions (Tomkins, 2016). Following the 2014 US military drawdown, drone strikes in Afghanistan accounted for 56% of weapons deployed by the US Air Force in 2015, up from 5% in 2011 (Reuters, 2016). Across Iraq and Syria, US drones have been integral to over 19,600 coalition airstrikes against Islamic State between August 2014 and April 2017 (Airwars, 2017). Predator and Reaper missions in Operation Inherent Resolve represent about a third of US Air Force missions, with approximately one in five drone sorties deploying a missile (Pawlyk, 2016). If these trends continue, US empire will maintain its domination in the third offset era not with a human-centered Baseworld but with a cyborgian *Roboworld.* The decline in large Main Operating Bases since the Iraq and Afghanistan occupations has been accompanied by a rise in small US bases or ‘lily pads’. Like Chabelley Airfield in Djibouti, these bases are often little more than runways for drones.

There are a growing number of bases across the globe integral to US military and CIA drone operations (Turse, 2012b). This Roboworld has materialized a drone surveillance and communications network that connects the planet via an electromagnetic rhizome. Countries that have been integral to US drone operations include: Afghanistan, Burkina Faso, Cameroon, Chad, Djibouti, Ethiopia, Germany, Italy, Iraq, Japan, Kenya, Kuwait, Niger, Pakistan, the Philippines, Qatar, Saudi Arabia, the Seychelles, Somalia, South Sudan, Turkey, Uganda, and the United Arab Emirates (the UK’s Menwith Hill and Australia’s Pine Gap bases both provide satellite information). Outside of these countries, Roboworld has also surveilled targets in Iran, Libya, Nigeria, and Yemen. More recently, the continent of Africa has become an important space of US air power. For the past decade, the US military has installed the scaffolding for a pan-continental aerial surveillance network. At the close of 2015, Pentagon officials began to release plans for a new, integrated system of drone bases to hunt Islamic State militants across Africa, South Asia, and the Middle East. This Roboworld is built of larger ‘hubs’, such as military bases in Afghanistan, with smaller ‘spokes’ constituted by lily pads, as with those in Niger (Mazzetti and Schmitt, 2015).

Roboworld aims to eradicate the tyranny of distance by contracting the surfaces of the planet under the watchful eyes of US robots. As Paul Virilio (1989: 59) argues, ‘This technological development has carried us into a realm of factitious topology in which all the surfaces of the globe are directly present to one another.’ Under a classic topographical spatiality, the boundaries of state-space coincide with physical locations, conforming to Max Weber’s classic definition of the state as a monopoly on the legitimate use of physical force within a territory. Here there is a Euclidian notion of insides and outsides, with state power housed in measurable, mappable, and distinct territories.

Topological space, in contrast, with its vocabulary of deformations, twists, cuts, and folds, signals the immanent, relational, and plastic spatial ontologies enabled by technology (see Martin and Secor, 2014). Topological thinking ‘draws attention to the spatial figures where insides and outsides are continuous, where borders of inclusion and exclusion do not coincide with the edges of a demarcated territory, and where it is the mutable quality of relations that determines distance and proximity, rather than a singular and absolute measure’ (Harvey, 2012: 78). The envelopment of the planet in a technological civilization has enabled all kinds of topological folds in communication, media, geopolitics, and, of course, warfare (Shaw, 2016). As Sloterdijk (2013: 139) writes, ‘thanks to radio-electronic systems, the meaning of distances has effectively been negated in the centres of power and consumption. The global players live in a world without gaps.’

The topological spaces of Roboworld thus deform the presumed link between sovereignty and territory, what Agnew (1994) labeled the ‘territorial trap’. Roboworld modulates and polices the planet’s surfaces. This capacity relies on a militarized code-space (Kitchin and Dodge, 2011) that ingests distant surfaces inside a computational ecumene. Accordingly, under a topological spatiality *relations determine distance*, rather than distance preexisting relations. As John Allen (2011: 284) argues, ‘power relationships are not so much positioned in space or extended across it, as compose the spaces of which they are a part’. The drone, by contracting great distances through robotic technics, has been productive of a series of lethal time-space compressions. ‘Empire presents a superficial world’, write Hardt and Negri (2000: 58), ‘the virtual center of which can be accessed immediately from any point across the surface.’ We can thus reinterpret Weber’s classic definition of the state not simply as the exercise of force *within* a territory (a topographic power), but as the control of distance *between* spaces (a topological power). US empire will continue to police this topological matrix. Such a robotic imperium is, however, largely a *one-way connection.* US drone pilots can strike distant targets, but the reverse is not true: a technological asymmetry still lies within empire.

‘By 2020’, argues Alfred McCoy (2012: 34), ‘the United States will deploy a triple-canopy aerospace shield, advanced cyberwarfare, and digital surveillance to envelop the earth in a robotic grid capable of blinding entire armies on the battlefield or atomizing a single insurgent in field or favela.’ Yet materializing such a planetary grid demands an infrastructure capable of going beyond the spatial topologies of human control. It requires the artificial intelligence of robots themselves.

## New spaces of empire III: The autogenic battle-site

Drone warfare has constructed remote power topologies that bridge human pilots with remote targets. Future autonomous drones, however, will collapse targets within robotic topologies, materializing a battlespace in which humans are *on* the loop, but not necessarily *in* the loop. The aleatory circulations of the warscape would be managed by an autonomous and adaptive system of robotic power. Future robotics, in short, challenge the human intentionality of state violence. Accordingly, we must anticipate not only the ongoing shift from topographic to topological spatialities, but also the transformation from remote to *autogenic* sites of power. ‘Autogenic’ comes from the Greek word meaning ‘self-generated’, or ‘coming from *within* the body’. Here, I use it to signal how robots will autonomously *generate* targets from within the technical body rather than directly respond to human directions. This artificial intelligence erodes human intentionality as the sole arbitrator of state power, replacing it with the machine learning of robotics. In doing so, it challenges our conceptions of biopolitics.

Biopolitics, what Foucault (2003: 240) called the ‘State control of the biological’, is oriented by the fear of anything, anyone, or anywhere *becoming-dangerous.* The US-led ‘war on terror’ mobilized this ‘kind of low intensity but all-pervasive terror of contingency’ (Dillon, 2007: 8). In Afghanistan and Iraq, the US military relied heavily on computation, biometrics, and GIS systems to manage this emergent quality of life, which was understood to possess ‘autonomous powers of adaptation, organisation and spontaneous emergence’ (Dillon and Reid, 2001: 55). Crucially, subjects of US security were rendered legible by algorithmic technics, which ‘radically subverts security’s traditional problematisation of pre-formed bodies operating in mechanical processes’ (Dillon and Reid, 2001: 56). Digital *information* became the means and ends for state power. This technically infused biopolitics does not target individuals per se, but the emergent becomings of what Deleuze (1992) once called *dividuals*: streams, patterns, and profiles of digital information. Life is no longer interpellated and secured via preformed bodies, or friend–enemy distinctions, but by a cybernetic war machine. Signature strikes, for example, are aerial bombings directed not towards known individuals but towards the weaponized time-space trajectories of dividuals (Shaw, 2016).

The emergent becomings of an aleatory life, in which the organic and inorganic collapse into a robotic information regime, transforms the battlespace. As discussed in the previous section, the militarized code-spaces of Roboworld materialize a shift from a Euclidian geometry, ‘where space is a homogenous plane’, to the topological spaces of ‘plural and complex spatial arrangements’ (Dillon and Reid, 2001: 55–56). Space is not a neutral plane of existence, a container for people and things, but a disruptive force-field: an emergent condition by which the nonhuman is agential and active, rather than passive and dead. How might this affect the battlespace?

Since the 1990s, the military has used the term ‘battlespace’ to describe a ‘full spectrum’ environment constituted by land, air, sea, space, friendly and enemy forces, the weather, the electromagnetic spectrum, and information. This notion of the battlespace underpins what Derek Gregory calls an everywhere war, where it is no longer ‘clear where the battlespace begins and ends’ (Gregory, 2011: 248). Yet, despite these expansive geographies, the battlespace maintains Cartesian separations between subjects and objects, evacuating the complex and agential interfaces between human and nonhuman bodies (Holmqvist, 2013; Wilcox, 2017). War is still understood, in the last instance, as a human condition. The possibility that nonhumans can generate and transform the battlespace is foreclosed.

Yet the reenchantment of the warscape through autonomous robots, ubiquitous sensors, and artificial intelligence, together with the algorithmic transformation of worlds (Amoore and Raley, 2017), is transforming the who, the what, and the how of the battlespace. Accordingly, we require an ontology that frames the battlespace as a more-than-human and emergent milieu, rather than a container of military action. Caroline Croser (2007) outlines the idea of an ‘event-ful’ battlespace in her study of US military strategy in Baghdad. To aid battlespace awareness in a complex urban insurgency, US commanders installed a GIS and GPS visualization program called Command Post of the Future. Crucially, the software mapped the city not as a static space of objects, but as an *event-ful space.* This technological interpellation produced a new form of Military Reason: a shift from a discrete target ontology of coordinates and objects (i.e. buildings or tanks) to an event-ful battlespace of *bodies-in-motion* and *becoming-dangerous*. For Derek Gregory (2010: 269), ‘the standard issue object-ontology was overwritten and increasingly overridden by an event-ontology: Baghdad was transformed into an event-ful city’.

**Figure 4.**
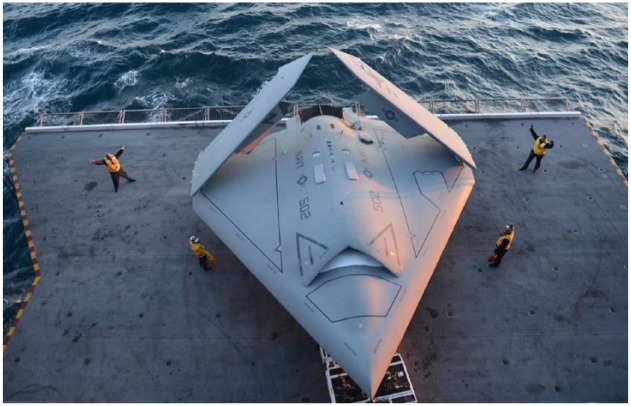
In 2013, the US Navy’s X-47B (pictured) became the first autonomous drone to land on an aircraft carrier. Photo by Timothy Walker. *Source*: Wikimedia Commons: *https://en.wikipedia.org/wiki/File:U.S._Sailors_move_a_U.S._Navy_X-47B_Unmanned_Combat_Air_System_demonstrator_aircraft_onto_an_aircraft_elevator_aboard_the_aircraft_carrier_USS_George_H.W._Bush_(CVN_77)_May_14,_2013,_in_the_Atlantic_Ocean_130514-N-FU443-025.jpg.*

How, then, can we understand the process by which the hybrid ecosystems of Roboworld will execute and manage conflict autogenically? The eventful battlespace of the future will be secured, transformed, and destroyed, by robotics. ‘Cooperative, autonomous systems can operate as self-healing networks and self-coordinate to adapt to events as they unfold’ (Scharre, 2014: 49). For centuries, technics has filtered, coded, and monitored biological existence. In the age of robotics, this artificial horizon is dissolving into millions of robots that can act autonomously: executing their own security, producing their own techno-geographies, and performing their own robotic being-in-the-world. Rather than being directed to targets deemed *a priori* dangerous by humans, robots will be (co-)producers of state security and non-state terror. The age of robots is the age of deterritorialized, agile, and intelligent machines.

We thus need a cartography for understanding the battlespace as robotic, autogenic, and oriented by the event. To realize this vision requires moving away from the ontological baggage associated with realist definitions of space, which typically understand it as a Newtonian container or a Cartesian extension. *Site*, alternatively, is a spatial concept that foregrounds the ontological singularity and autonomy of the more-than-human event. Sallie Marston et al. define the site as ‘an *emergent* property of its interacting human and non-human inhabitants’ (Marston et al., 2005: 425, emphasis in original). As they add, ‘a site ontology provides the explanatory power to account for the ways that … [a] collection of objects – can come to function as an ordering force in relation to the practices of humans’ (Marston et al., 2005: 425). Crucially, a site is ontologically autonomous, ‘the emergent product of its own immanent self-organization’ (Woodward et al., 2012: 214).

Accordingly, a site ontology advances our conception of military violence from that of a disenchanted battlespace overdetermined by human consciousness to that of an emergent *battle-site*, in which the robot is a co-producer of the event. The battle-site, like the battlespace, is a zone of conflict. Yet a battle-site is not an *a priori*, homogenous space then populated by people and robots, but is immanently generated by the synthesis of objects and subjects, of real and artificial intelligences. Battle-sites are immanent, dynamic, self-organizing event-spaces composed of more-than-human bodies and performances. They are, in other words, emergent singularities materialized by the synthesis of organic and inorganic forces. Autonomous robotics are transforming the transcendental conditions of state violence: a robotizing US empire emerges in and through the securitization of these battle-sites.

If human consciousness, emotion, affect, and flesh once directed the transcendental worldliness of the battlespace, then the battle-site is ordered by the *matter-processing* of robots. Matter-processing is the process by which robots and humans ‘co-think’ to actualize a set of virtual potentials and police the ontopolitical composition of worlds. As Woodward et al. write, ‘the affectivity not of people, but of *matter itself* – is an *immanent and autonomous* (that is, “self-legislating”) condition’ (Woodward et al., 2012: 211, emphasis in original). The battle-site is not a transcendent order imposed by human will, but the contingent outcome of a more-than-human matter-processing and machine learning.

In his discussion of drone warfare, Chamayou (2015: 52), argues that ‘armed violence is no longer defined within the boundaries of a demarcated zone but simply by the presence of an enemy-prey who, so to speak, carries with it its own little mobile zone of hostility’. Military drones continue to materialize a ‘temporary autonomous zone of slaughter’ (Chamayou, 2015: 55). As Chamayou (2015: 56) adds, ‘Depending on the contingencies of the moment, temporary lethal microcubes could be opened up anywhere in the world if an individual who qualifies as a legitimate target has been located there.’ The battle-site is similarly an emergent microcube of state violence: only, instead of being directed by the remote power of human pilots, it is generated by the artificial intelligence of robots.

In sum, autonomous robots will materialize new, non-Cartesian cartographies of autogenic state violence. This moves us from the discrete battlefields of *old wars*, the event-ful battlespaces of *new wars*, to the robotic battle-sites of *swarm wars.* These future conflicts will be realized by battle-sites of robotic violence in which organic and inorganic intelligence collapse in the violent machinations of matter-processing. This would materialize a US empire that extends beyond territories and bases to the deterritorialized robo-mesh of everyday life. Robotic sensors, robotic algorithms, and robotic swarms, resonating across the folds and surfaces of the city, would act together in a robotic manhunt, targeting the abstract trajectories of dividuals. Freed of the decidedly human calculations of goals, this ubiquitous robotic policing would materialize a post-political forever war, ‘a seamless, fluid, morphing and pulsating magma of conflict continuously forming and deforming according to changing impulses and instances of threat’ (Dillon and Reid, 2001: 63).

## Conclusion: A robot empire

The US military and the wider scientific community regularly imagine, construct, and script robot futurologies. These are not innocent or epiphenomenal projections: they must be thought of as *performative.* Robot futurologies prime and legitimize the conditions for investment, research and development, and state (in)security *now.* This article has itself constructed a futurology: one in which US empire, geopolitics, and warfare are fundamentally transformed by robotics. Its original contribution has been to pull a discrete set of imagined futures into the orbit of empire. In doing so, I do not assert this exact future will materialize – but, rather, a *set of conditions* that contain this future (and others like it) are now being imagined, researched, and materialized. Futurologies are non-linear and open to contingency. For this reason, it is vital that scholars, writers, activists, scientists, and artists intervene in the unequal geographies they mobilize.

Empires have risen and fallen with the evolution of land power, sea power, and air power. By analyzing the US defense community’s roadmaps, predictions, and anxieties, this article has explored the ascent of robot power and the potential futures of US warfare, geopolitics, and empire. The value of using empire across this article has been to constellate the past, present, and future, together with the spatial, the political, and the technical. Empire is not a spectacular monism: it is an uneven constellation of bodies and machines, neurons and algorithms, and the splintering warscapes of our more-than-human (in)security. Of course, robots possess a rebellious ontological quality (Walters, 2014) that disrupts easy predictions about the future. This uncertainty is compounded by the globalization of robots in militaries, militias, and terrorist groups across the world, which will transform the US-led robotic order of things. ‘This issue is particularly serious’, Kadtke and Wells (2014: 26) warn, ‘when one considers that in the future, many countries may have the ability to manufacture, relatively cheaply, whole armies of Kill Bots that could autonomously wage war.’

US empire is shifting, slowly, and never completely, away from a warfighting regime based on *human augmentation* – soldiers extending themselves as tool-beings – to an imperium of *robotic autonomy.* Swarms of autonomous robots are thus set to transform the spaces, logics, and agents of state violence. Graham Harman (2002: 220) argues we are ‘stationed unwittingly in a cryptic empire of force-against-force’. This cryptic empire of force-against-force can be understood as the material conditions of violence. From satellites, to IEDs, to Reaper drones, objects continually recondition an aleatory warscape. Empire is always immanent to this more-than-human matter-processing. The US military’s ultimate proxy war is composed of robots autogenically securing battle-sites, replacing humans as enforcers of empire. The possibility of materializing this kind of geopolitical future depends upon the US military’s ability (and resources) to install a Roboworld of artificial topologies across the planet. Contrary to the civilizing missions of 19th-century empires, US empire projects a capital-intensive vision of the future, an empire of robots nourished by the pervasive *techno-colonization* of the lifeworld.

This future involves the infiltration of empire into planetary technics: a robo-mesh. This would entrench a post-national and global biopolitics in which robots mediate the full spectrum of social interactions and perform a series of autogenic manhunts, collapsing separate spaces of state authority and violence. Future robotics, embedded across society, alongside ubiquitous sensing, computing, and the internet of things, would blur the boundaries between military, law enforcement, and commercial robotics, as well as everyday devices. ‘The profound ramifications of this trend are that nearly every aspect of global society could become instrumented, networked, and potentially available for control via the Internet’ (Kadtke and Wells, 2014: 46). We are only at the dawn of realizing such an expansive robo-mesh. The question, therefore, is not simply *how* robots will secure everyday life, but *who* they will secure. Robots could simply exacerbate and entrench preexisting conflicts and social inequalities.

Above all, a robotic US empire crystallizes the conditions for an unaccountable form of violence. By outsourcing the act of killing to artificial agents, violence – at least from the perspective of the US military – would transmute into an engineering exercise, like building a bridge, planned and executed by robots. In this sense, robot warfare is alienated because the act of violence functions without widespread human input. As one US Department of Defense-funded study predicts, ‘in the longer term, fully robotic soldiers may be developed and deployed, particularly by wealthier countries’ (Kadtke and Wells, 2014: 48). Such an ascendancy of robot soldiers embodies the triumph of *technics* over *demos.* As Tom Engelhardt (2012) argues, ‘think of us as moving from the citizen’s army to a roboticized, and finally robot, military…. [W]e are moving toward an ever greater outsourcing of war to things that cannot protest, cannot vote with their feet (or wings), and for whom there is no “home front” or even a home at all’. The risk here is that democracy abstains from the act of killing: on the loop, but no longer in the loop. An empire of indifference fought by legions of imperial robots.
